# Appendicular Soft Tissue Sarcoma Surgery in the Era of Orthoplastics

**DOI:** 10.3390/cancers18101578

**Published:** 2026-05-13

**Authors:** Inês Leitão, Joaquim Soares do Brito, Miguel Esperança-Martins, Cecília Melo-Alvim, Raquel Lopes-Brás, Daniel Jordão, André Abrunhosa-Branquinho, Filomena Pina, Dolores Lopez-Presa, Luís Vicente Saraiva, Arielle Turpin, José Portela

**Affiliations:** 1 Department of Plastic Surgery, Unidade Local de Saúde Santa Maria, 1649-028 Lisboa, Portugal; ines.amaro@ulssm.min-saude.pt (I.L.); 23019@chln.min-saude.pt (L.V.S.); arielle.turpin@ulssm.min-saude.pt (A.T.); 2 Department of Orthopedics, Unidade Local de Saúde Santa Maria, 1649-028 Lisboa, Portugal; jotaportela@gmail.com; 3 Department of Medical Oncology, Unidade Local de Saúde Santa Maria, 1649-028 Lisboa, Portugal; miguelmemartins@campus.ul.pt (M.E.-M.); cecilia.alvim.moreira@gmail.com (C.M.-A.);; 4 Luís Costa Lab., Instituto de Medicina Molecular–João Lobo Antunes, Faculdade de Medicina de Lisboa, 1649-028 Lisboa, Portugal; 5 Department of Surgery, Unidade Local de Saúde Santa Maria, 1649-028 Lisboa, Portugal; daniel.jordao@ulssm.min-saude.pt; 6 Department of Radiotherapy, Unidade Local de Saúde Santa Maria, 1649-028 Lisboa, Portugalfilomena.pina@ulssm.min-saude.pt (F.P.); 7 Department of Pathology, Unidade Local de Saúde Santa Maria, 1649-028 Lisboa, Portugal; doloreslopez@chln.min-saude.pt

**Keywords:** soft tissue sarcoma, multidisciplinary management, orthoplastic approach, collaborative surgery

## Abstract

Appendicular soft tissue sarcoma (aSTS) is a group of highly heterogeneous tumors of mesenchymal origin for which standard care usually includes surgical resection with or without radiation therapy. The main goal of surgical treatment in aSTS is to achieve wide tumor resection with negative margins while promoting the best function possible for the affected limb. Orthoplastic surgery represents the concept of a multidisciplinary approach comprising a synergistic collaboration between orthopedic and plastic surgeons. The development of this philosophy allows us to push forward the concept of limb salvage surgery for sarcomas, even when dealing with extremely complex cases. Reconstruction techniques integrated with orthoplastic principles range from simple to highly complex surgeries. Vascularized auto- or allo-graft tissue transfers illustrate how far reconstruction options can go, allowing for repair of large soft tissue defects or even restoration of muscle function in key anatomic segments after oncological resection. The reported experience with orthoplastic philosophy in aSTS suggests that it is a feasible and reproducible strategy that can achieve limb salvage rates above 90%, optimal oncologic local control characterized by more than 95% of wide margin resection, and improved functional and esthetic results. Most patients with aSTS treated under orthoplastic principles present good-to-excellent postoperative Musculoskeletal Oncology Society (MSTS) scores, confirming the advantages of this comprehensive approach. While there is significant experience with the orthoplastic approach for trauma cases, the road still needs to be paved for musculoskeletal oncologic reconstruction. Nonetheless, the results are promising and could inspire a wider adoption of structured orthoplastic protocols for sarcoma patient care. Herein, the authors explore the current practice regarding the application of collaborative orthoplastic approaches for the management of appendicular soft tissue sarcomas, reporting on outcomes and elaborating on future trends.

## Simple Summary

Appendicular soft tissue sarcoma is a group of highly heterogeneous tumors of mesenchymal origin for which standard care includes surgical resection and radiation therapy. The main goal of surgical management is to achieve wide tumor resection with negative margins while preserving the affected limb. The orthoplastic surgical approach represents the concept of a multidisciplinary synergistic collaboration between orthopedic and plastic surgeons. The development of this philosophy allows us to push forward the concept of limb salvage surgery for sarcomas, even when dealing with extremely complex cases, presenting limb salvage rates above 90%, good oncological control, and functional results ranging from good to excellent for most patients.

## 1. Introduction

Although soft tissue masses are an extremely common finding, sarcomas encompass a heterogeneous group of rare malignant neoplasms with mesenchymal differentiation that most commonly occur in the extremities and retroperitoneum [1]. Given their broad histological subtypes and their rarity, sarcomas are difficult tumors to approach, often presenting a dismal prognosis, with late diagnosis and advanced disease at presentation being common [2].

The management of soft tissue sarcomas represents a complex challenge requiring a multidisciplinary approach to optimize patient outcomes [3]. Historically, the treatment paradigm for extremity malignancies involved amputation, but advances in chemotherapy, radiotherapy, and surgical techniques allowed for a shift focused on limb-sparing strategies [4]. However, the anatomical location and size of sarcomas often imply extensive excisions that result in significant defects, both functional and esthetic in nature. Consequently, the difficulties associated with sarcoma resection and limb-sparing surgery are significant.

Orthopedic and plastic surgeons very often work separately, with the former addressing bone problems and the latter soft tissue deficiencies, creating an artificial separation and an unnecessary treatment gap between these surgical specialties. The synergistic integration of orthopedic and plastic surgical principles became known as the orthoplastic approach and has emerged as a powerful strategy for aSTS patients, aiming for optimal oncological control while improving limb function and esthetic outcomes and minimizing complications [5]. This philosophy allows us to push the boundaries of limb salvage surgery further, even in the presence of exceptionally complex cases. The collaborative strategy provided by the philosophy of orthoplasty represents a breakthrough in facing soft tissue sarcomas, mainly when optimal oncological resection implies complex soft tissue (and even bone) reconstruction [6,7,8].

Herein, the authors review the concepts and outcomes of the orthoplastic surgical approach applied for appendicular soft tissue sarcomas, discussing current management strategies and applications and elaborating on the future role for this philosophy to be integrated into the multidisciplinary care for sarcoma.

## 2. Principles of Orthoplastic Surgery Applied for Soft Tissue Sarcoma

The term orthoplasty was firstly introduced by Scott Levin in 1993 [9]. This author soon recognized the remarkable benefits of careful intraoperative soft tissue handling and timely reconstruction for bone and/or soft tissue defects with vascularized tissues [10,11,12]. As such, this approach can be applied to sarcoma management, embodying a multidisciplinary philosophy that integrates the orthopedic oncology law of complete tumor resection with negative margins as an absolute priority and plastic surgery’s ability to offer bone and soft tissue reconstruction and looks into superior functional and esthetical outcomes [7,13].

**Figure 1:**
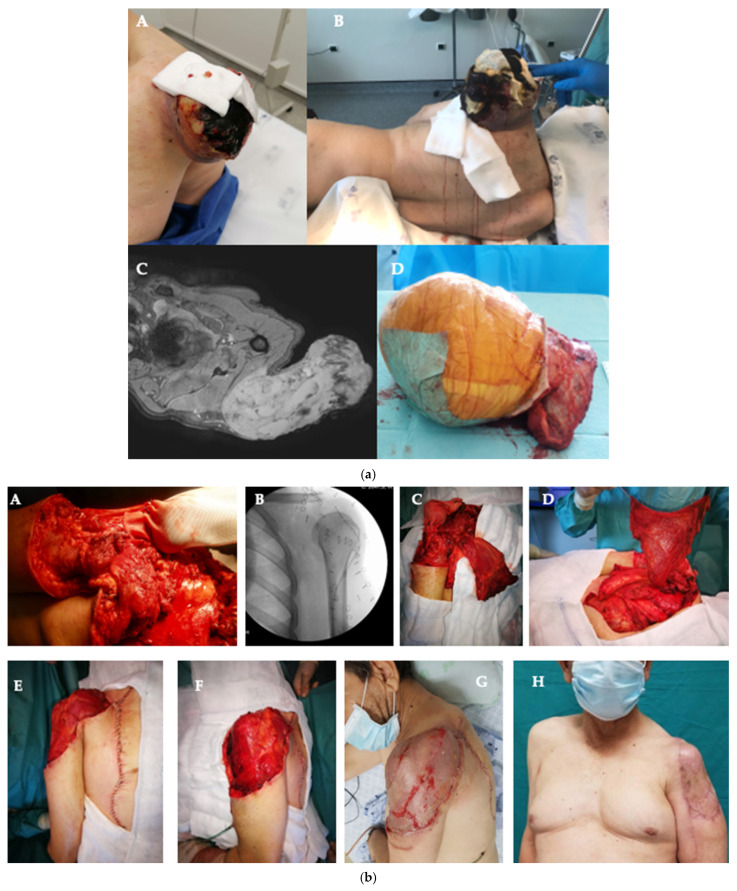
(**a**): (**A**)—Clinical photograph of a patient presenting an ulcerated undifferentiated pleomorphic sarcoma of the peri-scapular area; (**B**)—Clinical photograph of the same patient positioned in the operating room three weeks later and before surgical resection; (**C**)—Axial pre-operative MRI imaging showing the significant volume and extension of the sarcoma; (**D**)—Surgical specimen immediately after resection. (**b**): (**A**)—Intra-operative clinical photograph of the bone and soft tissue defect after the sarcoma resection. The humeral head is covered by a Trevira^®^ tube for further attachment to the remaining clavicle; (**B**)—Intra-operative radioscopic imaging after the sarcoma resection and showing the humeral head suspended with the Trevira^®^ tube into the remaining clavicle; (**C**)—Intra-operative clinical photograph showing the humeral head attached into the clavicle with the Trevira^®^ tube and a pedicled *latissimus dorsi* flap; (**D**)—Clinical intra-operative photograph presenting the *latissimus dorsi* flap; (**E**,**F**)—Clinical intra-operative photographs showing the result obtained after the coverage provided by the *latissimus dorsi* flap. A partial thickness skin graft was used on the muscle flap; (**G**)—Clinical photograph of the result approximately 2 weeks after the surgical procedure. (**H**)—Clinical photograph of the result (3 months after the surgical procedure).

The concept of negative margins is paramount in sarcoma surgery, although the definition of adequate margins can vary based on tumor grade, size, and location. Wide resection remains the standard approach regardless of the defect and disability it may entail, and marginal resection should be completely avoided due to the increased risk of local recurrence [14]. As such, the orthoplastic philosophy is particularly valuable in complex cases involving large tumors, neurovascular involvement, or extensive soft tissue defects, ensuring skeletal stability, optimized limb function, and durable soft tissue coverage [13] (Figure 1a,b).

## 3. Reconstruction Strategies

Reconstruction following oncologic excision must be individualized, considering the type of sarcoma, the anatomical location, the defect volume, the regional vascularity, prior radiotherapy, comorbidities, and the patient’s functional goals (Table 1) [14]. In addition, multidisciplinary discussion is of extreme importance to choose the optimal ablative or reconstructive approach. With these principles in mind, imaging studies are often necessary to estimate the size of the defect after excision with margins and possible reconstructive techniques.

When considering a reconstructive approach, we must consider two terms, “reconstructive ladder” and “reconstructive elevator”, which are key concepts in guiding choices for the best technique for wound coverage, tissue reconstruction, and functional/esthetic restoration [15,16].

The reconstructive ladder is a traditional model that organizes reconstruction options from the simplest to the most complex. The classic order of the ladder is as follows: healing by secondary intention, primary closure, skin grafts, local flaps, regional flaps, and microsurgical free flaps. The idea behind this concept is that the surgeon should climb the ladder gradually, choosing the least invasive solution that is sufficient to solve the defect [17]. This idea can be applied in situations where the simplest solution is adequate, facing small or moderate defects, and whenever managing patients with comorbidities that make complex surgeries unsuitable.

The reconstructive elevator is a more modern concept and does not require starting with simple techniques. This philosophy allows us to jump directly to the most appropriate technique, even with high complexity (i.e., microsurgical free flap), if that is the most appropriate strategy for the patient [18,19]. In complex defects that clearly will not be well managed by simple methods, the reconstructive elevator points towards the best possible functional and esthetic result, especially in complex scenarios.

While advanced microsurgical techniques are often necessary for massive defects, the orthoplastic approach must prioritize the simplest viable method to minimize donor-site morbidity and operative time. This aligns with the concept of the reconstructive elevator, as proposed by Gottlieb and Krieger [18]. Unlike the traditional ladder, which suggests a linear progression, the elevator allows the surgeon to select the most efficient technique—whether simple or complex—based on the specific “floor” (requirement) of the defect, while defaulting to simplicity whenever possible [19].

### 3.1. Secondary Intention

This simple strategy allows the wound to heal on its own, through granulation, contraction, and epithelialization. These principles can be applied when dealing with small and superficial wounds, patients with high surgical risk, and areas of low skin tension. The main advantages of this option are its simplicity, being non-invasive and to the simplicity of monitoring the involved area. The disadvantages are that, the longer healing time, the greater the risk for poor quality of scarring, and the greater the discomfort during healing. It can be unsightly in visible areas.

### 3.2. Primary Closure or Skin Grafting

This approach is only suitable for limited defects. Split- or full-thickness skin grafts may serve as temporary or definitive solutions, especially in areas with minimal mechanical stress. However, in irradiated zones or where deep structures are exposed, their use is limited due to poor vascularization and high failure risk. The advantages of using primary closure or skin grafting include being quick and simple, providing excellent esthetic results in most areas, allowing more predictable healing, and reduced cost and morbidity. The main disadvantages are not being applicable to large defects; the ability to generate excessive tension, which is associated with a risk of marginal necrosis or dehiscence; the requirement for an adequate vascular bed; and the fact that the color and texture may not correspond to those of the recipient site.

### 3.3. Minimalist Approaches and Skin Traction

In non-critical defects, particularly in elderly patients or those with significant comorbidities, minimalist techniques can avoid the risks of microvascular surgery:

○ Dynamic Skin Closure Systems: The use of skin traction devices allows for gradual wound closure over several days. This is particularly useful in irradiated fields where primary closure under tension would inevitably lead to dehiscence. By utilizing the viscoelastic properties of the skin (mechanical creep and biological stretch), these systems can often close defects that would otherwise require a flap.
○ Dermal Substitutes: Products such as Integra^®^ provide temporary coverage and promote the formation of a neodermis. This approach is an excellent alternative for patients who cannot tolerate long operative times, allowing for a delayed, simple skin graft once the wound bed is stabilized.

### 3.4. Local Pedicled Flaps

**Figure 2:**
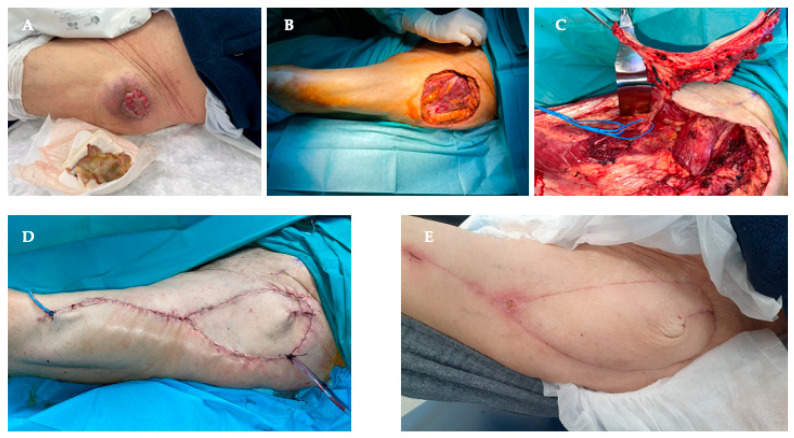
(**A**)—Clinical photograph of a patient presenting an ulcerated undifferentiated pleomorphic sarcoma of the left gluteal area. (**B**)—Intra-operative clinical photograph immediately after the sarcoma resection, showing the soft tissue defect. (**C**)—Anterolateral Tigh (ALT) pedicled flap being harvested. (**D**)—Intra-operative photograph showing the result with the ALT flap in place. (**E**)—Clinical photograph showing the result four weeks after surgery.

These flaps retain their native vascular pedicle and are transposed to the defect site, presenting a good vascularization with high success rate. The main advantages include shorter operative time and lower donor-site morbidity. They are particularly useful for small- to medium-sized defects where the adjacent tissue is healthy. In addition, these flaps allow for coverage of the defects with esthetics like the original tissue due to the proximity of the donor and recipient sites [14]. This aspect allows for great similarity in thickness, color, and texture (Figure 2). They maintain local sensation and functionality. A factor that may prevent successful flap transposition is the flap’s arc of rotation.

○ Reduced Morbidity: By using “nearby” tissue, the surgeon reduces the systemic physiological demand and avoids the complexities of finding recipient vessels outside the zone of injury or radiation.
○ Efficiency: Local pedicled flaps significantly reduce operative time compared to free flaps (like ALT or *latissimus dorsi*) while providing robust, vascularized coverage that is often sufficient for most post-sarcoma resections.

Examples of local pedicled flaps include the superior gluteal flap for sacral defects and the medial gastrocnemius flap for knee reconstruction. These flaps may be contraindicated in specific situations, particularly in trauma cases, where the extent and characteristics of the injury affect the viability of the surrounding tissues [20].

### 3.5. Regional Flaps

These flaps are typically located near but not immediately adjacent to the defect. They can be pedicled with a recognized vascular axis (e.g., pectoralis major flap, deltopectoral flap). The main advantages of this surgical option are the ability for greater reach than local flaps, the ability to cover large or deep defects, and the ability to bring well-vascularized tissue to compromised areas. Disadvantages include a more extensive surgery, greater morbidity in the donor area, the possibility of requiring multiple surgical procedures, and the potential for excessive tissue volume. This type of flap is most used for extensive defects that cannot be covered by local flaps, or for exposed bone, tendon, prosthesis, or other orthopedic implants [20,21].

### 3.6. Free Flaps

A free flap is defined as a segment of tissue that is completely removed from its original donor site and transplanted to a recipient site. This segment may include skin, subcutaneous tissue, muscle, or bone, along with its vascular pedicle. This procedure is made possible through an anastomosis between the flap vessels and vessels within the recipient site area, ensuring the viability of the transplanted tissue [22,23,24,25].

Free flaps have the great advantage of being able to be designed in detail according to the defect to be covered. The main advantages are maximum versatility (skin, muscle, bone, and all combinations of the three elements), allowing for highly complex reconstructions with excellent perfusion and reliability and better functional and esthetic results in many scenarios. In addition, they can restore precise volumes and contours. This type of reconstruction is important when we have an irradiated area, which makes coverage with local or regional patches impossible. Nonetheless, there are disadvantages, such as the long surgical time and need for an experienced team in microsurgery and advanced resources, together with the risks of microvascular thrombosis and greater physiological demand for the patient [22,23,24,25].

This type of reconstructive choice should be applied when we are dealing with large, deep, or complex defects and extensive exposure of critical structures.

#### 3.6.1. Muscle Free Flaps

Muscle free flaps are often used because they have large pedicles, are simple to raise, and present great ability to cover large defects [26,27]. These flaps provide better conformation to any irregular surface within the defect after sarcoma excision or to the orthopedic implants used in cases of bone fixation. They are also useful for cases where radiation therapy was used pre-operatively or planned in the post-operative period, avoiding pseudarthrosis, fractures, and bone or implant exposure [28,29]. Nonetheless, this type of flap has some disadvantages: the donor defect may lose some degree of function; the donor defect may be esthetically undesirable; reconstruction with muscle or musculocutaneous flaps may provide excessive bulk, leaving an esthetically unacceptable result; muscle or musculocutaneous flaps may atrophy over time and thus fail to provide adequate coverage; and removal of the muscle or musculocutaneous flap may result in contour deformities at the donor site. In addition, this type of flap can limit tendon gliding, making elevation for secondary surgeries more complex. The most used muscle free flaps are the *latissimus dorsi*, latissimus dentate, rectus abdominis, and *gracilis* muscle flaps [24,30,31].

The *latissimus dorsi* muscle is the largest muscle available and is an excellent option for covering extensive lesion areas, with exposure of tendons, nerves, and bone. Its dissection is relatively simple, and its pedicle is of reasonable length and caliber, making it a robust flap. Depending on the defect, it may be necessary to reposition the patient for flap harvesting [24].

The rectus abdominis muscle flap is a robustus flap, suitable for filling space in moderately sized deep wounds. However, donor site morbidity is a significant concern, with the risk of protrusion of abdominal contents and hernia formation [24].

The *gracilis* muscle has similar characteristics to those of the forearm muscles, with a long tendinous portion that is suitable for attachment to the finger tendons. For these reasons, this flap is extremely useful in restoring finger function with minimal donor site morbidity [30].

Functional innervated muscle flaps are also a good option. They allow restoration of active muscle function. *Gracilis* and innervated *latissimus dorsi* flaps have shown success in dynamic quadriceps and finger extensor reconstruction, with effective reinnervation reported in approximately 70% of cases [31,32,33,34].

#### 3.6.2. Fasciocutaneous Flaps

A fascial flap consists of fascia detached from its normal origin or insertion and transposed to another location. Without the overlying skin and fat, this represents a delicate flap. A fasciocutaneous flap, originally called an axial flap, includes the skin, subcutaneous tissue, and underlying fascia, which may be distinct from the fascia covering the underlying muscle [25].

The advantages include the following: they are thin and pliable, the blood supply is reliable and robust, the donor site morbidity is minimal in regard to function, they are muscle sparing, they have the ability to restore sensation, and there are many potential donor sites. There are disadvantages for this type of reconstruction: there is a lack of bulk for deep defects, they are technically more challenging (pedicle dissection; many require microvascular anastomosis or at least microvascular techniques), there are size limitations and the donor site may require skin graft closure, resulting in donor site deformity.

These characteristics have popularized the use of fasciocutaneous flaps as a first-line option in difficult upper limb reconstructions, where optimal coverage and early rehabilitation are crucial [25,35,36]. An example of an extremely versatile fasciocutaneous flap is the antero-lateral thigh (ALT) flap, which is based on the perforators vessels of the lateral circumflex femoral artery, which is a branch of the profunda femoris artery [25,37]. The ALT flap allows for filling deep defects and providing durable coverage, often in conjunction with bone grafts or prostheses.

#### 3.6.3. Osteotendinous and Nerve Reconstruction

When tendons or bones are resected, options include autologous or banked bone grafts, tendon grafts (e.g., palmaris longus, semitendinosus), or composite osteomuscular flaps (e.g., vascularized fibula). For nerve gaps greater than 3 cm, autologous nerve grafts such as sural or medial antebrachial cutaneous nerves are indicated.

#### 3.6.4. Vascular and Lymphatic Reconstruction

When major vessels are sacrificed, it is essential to immediately perform revascularization using autologous vein grafts (e.g., great saphenous vein) or, in selected cases, PTFE prosthetic grafts. Newer techniques include lymphovenous bypass for mitigating lymphedema following nodal dissection or radiation.

#### 3.6.5. Combined and Customized Reconstructions

In many cases—especially involving the trunk or shoulder girdle—hybrid solutions are required (e.g., muscle flap plus skin graft or free flap with bone prosthesis). 3D printing is increasingly used to design and fabricate customized implants for thoracic, pelvic, or scapular resections.

## 4. Orthoplastic Outcomes in Soft Tissue Sarcoma Surgery

Before the orthoplastic era, soft tissue sarcoma resection was usually followed by primary wound closure or simpler reconstructive methods. This strategy could be considered, even today, as the standard care for straightforward cases; however, whenever facing complex soft tissue defects after tumor resection, a simple closure can be challenging, or even impossible, to achieve. The emerging philosophy of orthoplastic surgery has allowed the pursuit of radical oncological clearance while preserving limb function, and a robust soft tissue coverage, which also translates into an improved quality of life [5,6,38].

Orthoplastic surgical management for soft tissue sarcomas is now a well stablished strategy. This philosophy has been proved to offer high limb-salvage rates with improved functional recovery, improved oncologic local control, and faster possibility to apply adjuvant therapies due to immediate definitive reconstruction (Table 2) [5,6].

Brown et al. reported that a combined oncoplastic approach (orthoplastic) allowed achievement of wide surgical margins in 97.4% of cases, which was higher than in non-combined oncoplastic groups, thereby contributing to better tumor control [39]. In addition, Götzl et al. published on the use of flaps (a key component of the orthoplastic strategy) as one factor minimizing the risk for local recurrence when compared with primary wound closure [40].

One of the main goals of applying orthoplastic principles is to minimize complications. Despite this being the goal, Davidge et al. could not find a significant difference in postoperative complication rates between patients receiving flap reconstruction (41%) and those with primary closure (31%); there is a clear tendency to use flaps and apply complex reconstruction principles only for complex defects [41]. As such, the lower complication rates after primary wound closure are more likely to represent simpler cases.

Angelini et al. reported that 38.5% of patients develop complications after single-stage resections and complex reconstruction under the orthoplastic philosophy, with the majority (90.3%) being minor and 10.4% experiencing flap loss [5]. Again, the cases where flaps were used had a higher level of complexity and where primary closure was not even possible to obtain. Cases where tumors have been preoperatively irradiated are another example of complex cases with a high level of complications to be expected [38]. As such, these types of cases are preferential clinical scenarios for the orthoplastic concept application.

Today, the orthoplastic approach is central to limb salvage surgery, presenting significant improvements in affected limb function. LaValley et al. found in their systematic review a 93.5% limb-salvage rate using the orthoplastic approach, with an 85.8% ambulation rate, and presenting a 21% rate for surgical review along the follow-up period [6]. In addition, Angelini et al. reported on the mean preoperative Musculoskeletal Tumor Society (MSTS) scores and Toronto Extremity Salvage Scores (TESSs), which significantly improved at final follow-up after an orthoplastic approach had been implemented (MSTS from 62.1 ± 23 to 74.8 ± 14; TESS from 69.4 ± 13 to 79.1 ± 13) [5].

Samà et al. reported on the results from a cohort with 211 patients with STSs where the orthoplastic approach was used. They estimate a 10% of cases where amputation was avoided due to the implementation of this surgical philosophy. In addition, extremely satisfactory functional outcomes were reported within this series [42].

**Table 2:** Some of the most relevant studies reporting on outcomes regarding the orthoplastic surgical approach for soft tissue sarcomas.

Study Year	Authors	Study Type	N	Surgical Technique	Key Outcomes
1999	Ihara et al. [43]	Case series	23	Reinnervated free muscle transplantation	All muscles grafts survived. One patient had partial skin flap necrosis and another had delayed healing after pre-operative radiation. Motor unit potentials were detected in all except one of the patients.
2010	Davidge et al. [41]	Case series	247	Flap reconstruction vs. primary closure	Postoperative complications occurred in 41% of patients receiving flap reconstruction vs. 31% of patients treated with primary closure. Most complications were wound-related.
2012	Grinsell et al. [34]	Case series	22	Innervated muscle flap reconstructions	Wide resection was completed in all the cases except for one. One patient had a superficial infection at the donor site. There were a total of 6 (28.5%) postoperative complications related to the reconstruction: 2 major and 4 minor. The 2 major complications were flap failures due to necrosis.
2013	Payne at al. [44]	Prospective	113	Pedicled vs. free flap (upper limb)	23% (26 cases) of the cases had a complication, which was equally divided between the two groups. Wound infections (12 cases) and delayed healing (7 cases) were the most common complications. There was one partial ALT free flap failure. Comparison of the post-operative MSTS and TESSs revealed no significant differences between the free- and pedicled-flap groups.
2018	Slump et al. [45]	Comparative	266	Pedicled vs. free flap	Post-operative surgical complications occurred in 90 (34%) of the patients. 52 complications were classified as major. No difference in complication rates between free or pedicled flaps (38% vs. 32%). There was no significant difference between functional outcomes between free or pedicled flaps in either the upper or lower limb reconstructions.
2020	Brown et al. [39]	Comparative	546	Combined oncoplastic approach (orthoplastic) vs. primary wound closure	Surgical wide margins were obtained in 97.4% of the cases using a combined oncoplastic approach. Local recurrence was 4.9% with the combined oncoplastic approach. An estimated risk reduction of 52% for local recurrence with the combined oncoplastic approach. Similar wound complication rate between combined oncoplastic approach and primary wound closure.
2020	Othman et al. [27]	Case series	49	Flaps after radiotherapy	16 (31.3%) flaps presented complications; wound complications included infection, dehiscence, hematoma, seroma, delayed wound healing and flap takeback. 11 flaps (21.6%) required reoperation; reasons for re-operation included infection, wound dehiscence, flap congestion due to arterial occlusion requiring revision with successful flap salvage in two occasions and two flap failures. Two patients suffered from complete flap loss. Peripheral vascular disease and diabetes were associated with wound complications; radiation was not associated with any complications.
2020	Angelini et al. [5]	Case series	161	Single-stage resection and complex reconstruction	38.5% of patients developed a complication (90.3% minor and 9.7% major). 10.4% flap loss. Significant improvements in limb function scores post-surgery: MSTS and TESS were 74.8 ± 14 and 79.1 ± 13, respectively.
2021	Lucattelli et al. [26]	Systematic Review	132	Upper extremities free flap	Only 3 cases of flap loss were reported among 132 patients receiving a free flap.
2022	Hassan et al. [33]	Meta-Analysis	70	Free-functioning muscle transfer	Flap loss rate was 0.8%. During the follow-up period, 31% of patients (4 cases) developed local tumor recurrence and 85% developed tumor metastasis. The median time to first neuromuscular recovery and maximal neuromuscular recovery was 1.5 months (range: 0.3–6.4 months) and 7.6 months (range: 2.3–23.8 months), respectively. A high BMI was associated with delayed first neuromuscular recovery.
2024	Lavalley et al. [6]	Systematic Review	1060	Lower extremity free and local flaps	Of 553 patients with specific outcomes provided, infection occurred at a rate of 11.9%, wound dehiscence at 8.0%, seroma formation at 5.8%, delayed wound healing at 5.6%, partial flap necrosis at 5.6%, total flap loss at 4.2%, and hematoma formation at 2.0%. 20.6% of patients required revision surgery during their follow-up. Limb salvage rate was 93.4%. 85.8% ambulated postoperatively. The most common functional scores reported were the Lower Extremity MSTS score, the TESS, and the Lower Extremity Functional Score, with mean scores of 26.8, 82.6, and 69.4, respectively.

Innocenti et al. reported excellent limb salvage and functional outcomes with *latissimus dorsi* transfers in quadriceps reconstructions, noting 73% good-to-excellent MSTS scores [31]. Grinsell et al., similarly, demonstrated successful single-stage innervated *latissimus dorsi* and *gracilis* free flaps in sarcoma cases, reporting an average MSTS score of 84% for the lower extremities and 71% for the upper extremities [34].

Overall, the favorable results reported when using the orthoplastic approach for STSs seem to be favorable to its use in a systematic fashion whenever needed. Nonetheless, the decision-making process for timing and sequencing for these interventions is highly individualized.

Soft tissue reconstruction success is consistently high (flap survival > 90% with flap loss in approximately 5% of cases), underscoring the reliability of microsurgical flaps in limb salvage surgery [6]. The ideal reconstruction strategy must balance surgical complexity, operative time, available resources, and, most importantly, the patient’s functional needs and life expectancy.

## 5. The Role of Multidisciplinarity in Soft Tissue Sarcoma Management

As a rare and genetically heterogeneous malignancy, soft tissue sarcomas pose substantial diagnostic and management concerns, requiring a coordinated strategy that goes beyond the orthoplastic philosophy for surgical approach [43]. As such, multidisciplinary board meetings are considered fundamental to maximize patient outcomes. This collaboration provides a composite of skills in surgical oncology, radiation oncology, medical oncology, pathology, and imaging, to create a tailor-made treatment plan depending on the specific histological variant of the tumor, location, and patient health. This integrated approach is crucial for navigating the complexities of soft tissue sarcomas, ensuring that all aspects of patient care, from initial diagnosis to long-term follow-up, are meticulously addressed [44].

An important aspect of such planning would be the careful consideration of both the radical tumor excision and the need for salvage of the limb. Neoadjuvant therapies, such as chemotherapy and radiotherapy, are commonly employed for downstaging, facilitating R0 resections, as well as enhancing local control, although the optimal role within surgical reconstruction strategies is still an ongoing discussion [45].

The importance of a centralized, multidisciplinary approach to soft tissue sarcoma management cannot be emphasized enough, as this approach dramatically improves diagnostic accuracy, treatment efficacy, and overall patient prognosis in this orphan and complex disease [46,47,48,49]. This approach ensures that each patient receives a comprehensive treatment plan that is attuned to the nature of soft tissue sarcomas, leading to more favorable long-term outcomes and a better quality of life [48,49]. Such specialized sarcoma centers, where these multidisciplinary teams are often housed, have shown improved patient outcomes, such as longer survival and lower recurrence rates. There is also evidence from European networks of an improvement in disease-free survival related to presenting sarcoma cases at a multidisciplinary tumor board and adhering to established standards of care [48,50,51].

## 6. Orthoplastic Management Protocol for Appendicular Soft Tissue Sarcomas

### 6.1. Preoperative Assessment and Risk Stratification

Multidisciplinary planning (Tumor Board) is mandatory to identify high-risk patients who require proactive intervention.

- *Wound Complication Risk Factors:*
  ○ Preoperative Radiotherapy: The strongest predictor of dehiscence, particularly in lower limbs.
  ○ Critical Location: Distal third of the tibia, foot, and periarticular areas (knee/ankle).
  ○ Patient Comorbidities: Active smoking, Diabetes, and BMI > 30.
- *Vascular Mapping:*
  ○ CT–Angiography or Doppler for large tumors or those involving major neurovascular bundles.

### 6.2. Decision-Making: The Reconstructive Elevator

Surgeons should utilize the “Reconstructive Elevator” to select the most efficient technique—prioritizing simplicity and proximity—rather than following a rigid linear ladder (Table 3).

### 6.3. Indications for “Skipping Rungs” (Immediate Complex Reconstruction)

Immediate free flap reconstruction is indicated when the following apply:

○ Major vessels are involved, requiring bypass or critical vascular protection;
○ Healthy local tissue is unavailable due to prior surgeries or extensive high-dose radiation;
○ Large dead spaces must be filled to prevent chronic seroma or infection.

### 6.4. Intraoperative Orthoplastic Principles

○ R0 Resection: Reconstruction must never compromise oncological margins;
○ Pedicle Preservation: Early identification of recipient vessels for potential flaps during tumor resection;
○ Rigorous Hemostasis: Prevention of hematomas that could compromise tissue transfer viability.

### 6.5. Postoperative Care and Rehabilitation

○ Selective Immobilization: Protecting anastomoses or sutures in joint areas;
○ Negative Pressure Wound Therapy (NPWT): Used as a bridge for “Delayed Reconstruction” or to secure skin grafts;
○ Early Physiotherapy: Initiated as soon as wound stability allows to optimize functional limb salvage.

## 7. Discussion

Soft tissue sarcomas represent rare tumors arising in mesenchymal tissues, which can occur almost anywhere in the body. Their rarity and heterogeneity mean that developing evidence-based guidelines is an extremely difficult task. As such, soft tissue sarcomas should be managed by expert multidisciplinary teams to ensure consistent and optimal treatment [51,52].

The orthoplastic approach emerged as a multidisciplinary paradigm shift in the surgical management of soft tissue sarcomas, emphasizing the integration of oncologic principles with reconstructive techniques to optimize patient outcomes. The collaborative synergy between orthopedic and plastic surgeons is paramount in addressing complex cases, ensuring comprehensive management that neither specialty could achieve in isolation [53,54,55,56,57]. The goal of this approach is to achieve complete tumor resection, with adequate safe margins, while minimizing functional morbidity and maximizing quality of life. The collaboration between orthopedic and plastic surgeons allows for comprehensive assessment and management of complex soft tissue defects, facilitating the use of advanced reconstructive techniques such as microvascular free tissue transfer and complex local flaps [58].

The existing literature supports the use of combined modalities, including surgery and radiation, for soft tissue sarcoma management, with limb-salvage surgery becoming the standard of care for extremity sarcomas, which presents comparable survival rates to amputation while offering good functional outcomes and quality of life [6,7,8].

Reconstruction options following sarcoma resection range from simple primary closure to complex free tissue transfer, depending on the size and location of the defect. Since local recurrence remains a significant challenge in soft tissue sarcoma management and is associated with poorer overall survival, the orthoplastic approach facilitates its management, aiming for optimal functional outcomes with low incidence of wound complications and need for secondary procedures [23,59,60,61,62].

It is important to understand that sarcoma patients differ from those with tissue loss due to trauma or infection, since they often present several comorbidities, poor nutritional status, and the need for adjuvant therapies, which will have a systemic impact and adverse effects on local tissue [5,63,64]. Flap reconstruction is therefore often necessary to decrease wound-healing complications and infection rates. The development of such complications may delay further medical treatment, and the extent of plastic reconstruction should be balanced between what the patient needs and what the patient can tolerate surgically within the context of overall treatment and survival prognosis [28,65].

The orthoplastic approach not only applies for skin, muscle, and bone defects, but also to nerve and vessels reconstructions. For most patients, a wide resection of the sarcoma will be possible without interfering with major vessels or nerves; however, for those rare cases where vascular or nerve resections will be needed, subsequent reconstruction to enable limb salvage is the rule [60]. Major nerve resection can be reconstructed with primary nerve cable grafts, nerve transfers or even tendon transfers [6,7]. As for vascular reconstructions, bypass with autograft or synthetic graft will allow maintenance of the distal vascularity of the limb. Lymphatic–venous anastomosis for treatment of the extremity after cancer treatment is a recent surgical advancement with growing applications [6,7].

As the multidisciplinary surgical management of soft tissue sarcomas becomes widespread, benefits are more often reported. The orthoplastic synergistic strategy has proven to lead to lower complication rates, fewer secondary procedures, and shorter hospital stays, with an improved limb-salvage rate. While specific protocols for complex soft tissue reconstruction integrated on an orthoplastic philosophy are lacking, this collaboration between orthopedic and plastic surgeons seems to be essential to improve patient outcomes.

### Innovations, Gaps, and Future Directions

Despite all merits attributable to the orthoplastic philosophy, whenever approaching soft tissue sarcomas, there are gaps to be filled. In this regard, the recent integration of artificial intelligence (AI) in preoperative surgical planning, surgical procedures, and postoperative care is a novelty that can help to close the gap [66]. This AI use seems promising when dealing with aSTS, helping to define a more precise workflow. This technology allows a more refined selection of the most appropriate reconstructive technique, helps on intra-operative decisions, can reduce operative time, minimizes human error during delicate procedures, and promotes more accurate post-operative monitoring [67,68]. Additionally, machine learning algorithms can predict post-operative complications, which facilitates the prediction of further interventions for high-risk patients. Altogether, AI presents the potential ability to boost clinical performances when managing soft tissue sarcomas, enhancing personalized medicine practice to each clinical setting [69,70]. This scenario gains even more traction in this modern era of high medical-care demand combined with staff shortages. As such, it is highly predictable that a more common and widespread integration of AI technologies in soft tissue sarcoma approach will occur. The future is just around the corner.

## 8. Conclusions

The management of aSTSs remains a challenge. The emergence of the synergistic collaboration between orthopedic and plastic surgeons paved a new era in the surgical management of aSTS, particularly for complex cases. This new strategy, known as orthoplasty, allowed the limits on the ability to perform limb-salvage surgeries to be pushed.

Despite the giant step forward, much still needs to be accomplished. It is paramount to develop structured protocols and integrate new technologies for a systematic use of this surgical approach to potentiate the outcomes for our future patients.

## Figures and Tables

**Table 1:** Unidade Local de Saúde Santa Maria institutional protocol for reconstructive options according to defect and affected anatomical region.

Anatomical Areas	Reconstructive Options
Upper Limb	1. Primary closure/grafts Small defects, superficial burns 2. Local flaps Advancement and rotation flaps Island flaps (very commonly used in hand and forearm) 3. Regional flaps (Tendon, nerve, and bone coverage) Posterior interosseous flap Reverse radial flap Ulnar flap 4. Free flaps ALT *Gracilis* (functional reconstruction) *Latissimus dorsi* (extensive or traumatic defects)
Lower Limb	1. Skin grafts Superficial defects with a good wound bed 2. Local flaps Fasciocutaneous flaps Local muscle flaps 3. Regional flaps (bone and fixation material coverage) Gastrocnemius (knee) Soleus (mid-leg) Medial plantar 4. Free flaps ALT (anterior talofibular joint) *Latissimus dorsi* *Gracilis* (essential in the distal third of the leg and foot)

**Table 3:** Decision-making summary for best surgical technique to apply based on the Reconstructive Elevator principle.

Reconstruction Level	Technique	Main Indications	Clinical Benefit
Minimalist	Dynamic Skin Traction	Small to medium defects with skin tension; irradiated fields.	Avoids flap surgery; utilizes skin viscoelasticity.
Minimalist	Dermal Substitutes (e.g., Integra^®^)	Frail patients; exposed bone/tendon in non-weight-bearing areas.	Reduces operative time; avoids donor-site morbidity.
Local (Nearby)	Pedicled Flaps (e.g., Gastrocnemius, Soleus)	Defects in the knee or middle-third of the leg; healthy local tissue.	Reliable vascularity; simplified post-op monitoring.
Complex	Free Flaps (e.g., ALT, *latissimus dorsi*)	Massive defects; lack of local tissue; previous high-dose radiation.	Provides large volume of well-vascularized tissue.
Salvage	Cross-leg or Chimeric Flaps	Severe vascular depletion; extensive limb-threatening trauma.	Limb salvage in extreme scenarios.

## Data Availability

The raw data supporting the conclusions of this article will be made available by the authors on request.
